# “Now I can see it works!” Perspectives on Using a Nutrition-Focused Approach When Initiating Continuous Glucose Monitoring in People with Type 2 Diabetes: Qualitative Interview Study

**DOI:** 10.2196/67636

**Published:** 2025-01-10

**Authors:** Holly J Willis, Maren S G Henderson, Laura J Zibley, Meghan M JaKa

**Affiliations:** 1 International Diabetes Center HealthPartners Institute Minneapolis, MN United States; 2 Center for Evaluation and Survey Research HealthPartners Institute Bloomington, MN United States

**Keywords:** diabetic, diabetes mellitus, DM, type 2 diabetes, T2D, endocrinology, nutrition, diet, continuous glucose monitoring, glucose monitor, glucose, glycemic control, time in range, self-care, education, mHealth

## Abstract

**Background:**

Food choices play a significant role in achieving glycemic goals and optimizing overall health for people with type 2 diabetes (T2D). Continuous glucose monitoring (CGM) can provide a comprehensive look at the impact of foods and other behaviors on glucose in real time and over the course of time. The impact of using a nutrition-focused approach (NFA) when initiating CGM in people with T2D is unknown.

**Objective:**

This study aims to understand the perspectives and behaviors of people with T2D who participated in an NFA during CGM initiation.

**Methods:**

Semistructured qualitative interviews were conducted with UNITE (Using Nutrition to Improve Time in Range) study participants. UNITE was a 2-session intervention designed to introduce and initiate CGM using an NFA in people with T2D who do not use insulin. The intervention included CGM initiation materials that emphasized the continuous glucose monitor as a tool to guide evidence-based food choices. The materials were designed to support conversation between the CGM user and diabetes care provider conducting the sessions. A rapid matrix analysis approach was designed to answer two main questions: (1) How do people who participate in an NFA during CGM initiation describe this experience? and (2) How do people who participate in an NFA during CGM initiation use CGM data to make food-related decisions, and what food-related changes do they make?

**Results:**

Overall, 15 people completed interviews after completion of the UNITE study intervention: 87% (n=13) identified as White, 60% (n=9) identified as male, mean age of 64 (SD 7.4) years, mean T2D duration of 7.5 (SD 3.8) years, and mean hemoglobin A_1c_ level of 7.5% (SD 0.4%). Participants fluently discussed glycemic metrics such as time in range (percent time with glucose 70-180 mg/dL) and reported regularly using real-time and retrospective CGM data. Participants liked the simplicity of the intervention materials (eg, images and messaging), which demonstrated how to use CGM data to learn the glycemic impact of food choices and suggested how to adjust food choices for improved glycemia. Participants reported that CGM data impacted how they thought about food, and most participants made changes because of seeing these data. Many of the reported changes aligned with evidence-based guidance for a healthy lifestyle, including prioritizing nonstarchy vegetables, reducing foods with added sugar, or walking more; however, some people reported behavior changes, such as skipping or delaying meals to stay in the target glucose range. A few participants reported that the CGM amplified negative feelings about food or eating.

**Conclusions:**

Participants agreed that pairing nutrition information with CGM initiation instructions was helpful for their diabetes care. In general, the NFA during CGM initiation was well received and led to positive changes in food choices and behaviors during a 2-month intervention.

## Introduction

### Background

First-line therapy for the management of type 2 diabetes (T2D) is lifestyle modification, which includes following evidence-based nutrition and physical activity guidelines [1]. Food choices can play a significant role in achieving glycemic goals and optimizing overall health for people with T2D [2]. Moreover, continuous glucose monitoring (CGM) has also been shown to improve glycemic outcomes for people with T2D [3]. CGM can provide a comprehensive assessment of the impact of foods and other behaviors on glucose in real time and over the course of time. People with T2D may benefit from using CGM data to guide food choices that help achieve their desired glycemic goals, including time in range (TIR; percent time with glucose levels between 70-180 mg/dL).

However, people with T2D may encounter challenges with knowing how to use CGM data to make food choices, especially making food choices that can maximize TIR and that are good for overall health. In other words, it may not be clear which food choices keep glucose in the desired target range and align with current evidence-based nutrition guidance for people with diabetes [4].

Optimal CGM use requires education, training, and support [5]. Various tools [6], methods [7], and programs [8] have been created to educate CGM users on the effective use of CGM technology and its associated data. However, specific emphasis on evidence-based nutrition guidance has not been embedded into these trainings, and this could have consequences. For example, without nutrition guidance, a continuous glucose monitor could lead its user to regularly choose *less healthy* foods if those foods keep glucose in the target range of 70 to 180 mg/dL (eg, choosing high-fat red meats or highly processed low-carbohydrate snack foods); however, these *less healthy* foods may be detrimental to other aspects of health and lead to unintended consequences.

Research suggests that people who are empowered and skilled to self-manage their diabetes have improved health outcomes [9,10]. Discovery learning is one self-care opportunity, which has been described by Polonsky et al [11] as a time when an individual with diabetes is supported to make use of new information (such as one’s own glucose values) to gain insights through personal experience and reflection. Having CGM data available before and after meals can provide a profound opportunity for the user to make connections between a given glucose value and food choices, portions, or circumstances, which, in turn, could promote data-driven behavior changes. Thus, this suggests that evidence-based nutrition recommendations at the time of CGM introduction and initiation could be beneficial.

### This Study

The purpose of this research was to understand the perspectives and behaviors of people who participated in a nutrition-focused approach (NFA) when starting CGM. More specifically, this research in non–insulin-using people with T2D describes the following: (1) How do people who participate in an NFA during CGM initiation describe this experience (ie, intervention receipt)? and (2) How do people who participate in an NFA during CGM initiation use CGM data to make food-related decisions, and what food-related changes do they make (ie, intervention enactment)?

The outcomes of this research can help identify gaps in knowledge regarding how new CGM users understand and use their CGM data to make food-related decisions. This research can also provide the diabetes care community with considerations for how to present or position nutrition messages when initiating CGM in people with T2D.

## Methods

### Study Design

This qualitative study is part of the larger UNITE (Using Nutrition to Improve Time in Range) study (NCT05928572). UNITE is a randomized clinical trial designed to understand if there are differences in glycemia and dietary intake when people with T2D are introduced to CGM using 2 different methods. The 2 CGM initiation methods were an NFA and a self-directed approach (SDA). Participants were randomly assigned to participate in either the NFA or SDA when initiating a Dexcom G7 (Dexcom, Inc) CGM sensor paired with a smartphone app. All UNITE study participants used the G7 sensor and smartphone app continuously for approximately 2 months.

The focus of the NFA was to help CGM users use their CGM data to identify which food choices align with evidence-based nutrition recommendations and help achieve glycemic goals. Development of the NFA has been previously described by Willis et al [12]. In brief, the NFA included the following three components: (1) a 60-minute, in-person CGM initiation session; (2) a 30-minute, remote CGM data review session occurring approximately 14 days after CGM initiation; and (3) nutrition-focused CGM initiation materials designed to support both the CGM user and the diabetes care provider conducting the sessions. The materials included a brief interactive slide presentation containing graphic images and a 1-page CGM nutrition guide. The materials encouraged the CGM user *to know* their glucose goals (including a target glucose range of 70-180 mg/dL and TIR of >70%); *to learn* how their body responds to foods and activity using a *1, 2, 3* approach (a method for following glucose before and after meals and activity to learn the body’s response); and to consider how *to adjust* food choices using a *yes/less* framework (a highly simplified version of evidence-based nutrition recommendations). Excerpts of the materials and how they were used are published elsewhere [12]. A registered dietitian nutritionist served as the diabetes care provider for both sessions; however, the sessions were not intended to replace medical nutrition therapy (eg, the NFA did not include a full nutrition assessment or diagnosis). While a registered dietitian nutritionist would be an excellent candidate to deliver the NFA, the nutrition-specific content was developed to be general enough that other care providers could be trained to deliver the intervention. An intervention manual was used to keep the content and sessions consistent among all participants in the UNITE study.

A rapid matrix analysis approach with semistructured qualitative interviews [13,14] was designed to describe intervention receipt (this included information about the quality and quantity of information delivered and about the intervention materials, including the interactive slides and the CGM nutrition guide) and intervention enactment (this included thoughts and behaviors related to CGM use and food choices).

A deductive approach (ie, one that uses an existing framework to guide the qualitative coding process) [15] was selected because the National Institutes of Health fidelity framework [16] provided an appropriate a priori coding tree that could be applied to the NFA intervention. Constructs included a description of the participants’ diabetes history; intervention receipt, including interventionist and intervention materials; and intervention enactment, including CGM only (no food) and food with or without CGM. The qualitative study was designed and reported following the COREQ (Consolidated Criteria for Reporting Qualitative Research) guidelines [17].

### Ethical Considerations

All protocols and procedures for this qualitative study were reviewed and approved by the HealthPartners Institutional Review Board (study A22-279) in July 2023; this was approved before contacting participants. Verbal informed consent, as approved by the institutional review board, was obtained from each participant at the time of the interview. Participant confidentiality and privacy were maintained using the following methods: (1) study staff were trained in human subjects research protections and Health Insurance Portability and Accountability Act compliance, (2) any study-related data were collected and stored on password-protected servers behind a firewall to which only study staff had access, and (3) participant information was deidentified, to the extent possible, using numerical IDs. Participants who completed the interview received a US $25 Target gift card.

### Recruitment and Participants

Participants were eligible for the qualitative study if they met inclusion criteria for the larger UNITE study, were randomly assigned to the NFA arm, completed all components of the 2-month intervention, had adequate CGM data at the final postintervention assessment, and were willing to participate in a recorded interview. In brief, eligibility criteria for the UNITE study included being aged ≥18 years; a T2D diagnosis; having a hemoglobin A_1c_ (HbA_1c_) of 7% to 10% at the time of screening; having a stable diabetes medication regimen for at least 30 days excluding any form of insulin, sulfonylureas, meglitinides, or other medications with known hypoglycemia risk; and having no personal CGM use within 90 days before the start of the study.

Individuals who met screening criteria were asked by UNITE study staff via phone if they were interested in participating in a qualitative interview. If so, they were scheduled for a single 30-minute phone call that took place at the clinic after their final UNITE study visit. Only the participant and the interviewer were present during the interview. Participants were informed that they were speaking with a trained health care interviewer and that the purpose of the interview was to learn about their experience in the study to improve CGM initiation options in the future. To increase the likelihood of saturation in qualitative analysis [18], up to 15 interviews were planned, and an effort was made to balance the invitation of participants by gender identity.

### Data Collection

Phone interviews were conducted using an interview guide aligned with the a priori coding tree described earlier, starting with intervention receipt followed by enactment. The guide was developed by the research team (HJW, MMJ, MSGH, and LJZ; all identified as female) following the best practices for semistructured interviewing [19]. Interviews included a series of open-ended root questions with follow-up probes to elicit richer data from participants. The interview started with an easy-to-answer rapport-building question to set the tone and then funneled from broad to more specific questions, ending with a final cool-down question. During the intervention receipt portion of the interview, participants were asked to recall the intervention materials unprompted and were asked to look at copies of the materials to encourage more detailed recall. In the intervention enactment portion, participants were asked to describe how they used CGM data and how the data affected their thoughts about food, food choices, and eating behaviors. Interviewers were encouraged to probe for specific examples. The interview was designed to be completed within 30 minutes. Textbox 1 summarizes the interview questions. The full interview guide can be found in the Multimedia Appendix 1.

Interviews were conducted by trained qualitative interviewers (MSGH and LJZ) with master’s degrees in health-related fields and experience conducting interviews with participants in health care–related research studies. The interviewers were involved in previous qualitative research on CGM use by people with diabetes and diabetes care providers. Interviewers also received study-specific interview training from a diabetes researcher (HJW) and conducted practice interviews with diabetes care and education specialists. Ongoing supervision by a qualitative researcher (MMJ) was provided to prevent drift in facilitation over time.

**Interview guide summary, including question purpose, summarized interview questions, and probes.**

**Rapport building**

- What do you remember about when you were first diagnosed with diabetes?
  How did you take care of your diabetes at that time?
  Did you think about nutrition or food choices at that time?
  Did you ever talk with a diabetes educator or dietitian? Tell me about that experience.

**Intervention receipt (how do people with type 2 diabetes who participate in a nutrition-focused approach during continuous glucose monitoring [CGM] initiation describe their experience?)**

- What do you remember talking about with your diabetes care provider when you first started using your CGM?
  What did you think about the nutrition-focused information you received and how it was presented?
  What did you like (or what could be improved) about the materials that were used to help you learn to use your CGM? (this question was asked unprompted and prompted)
  Do you think focusing on nutrition (food choices) is a good way to help someone get started using their CGM? Why or why not?

**Intervention enactment (how do people who participate in a nutrition-focused approach during CGM initiation use CGM data to make food-related decisions and what food-related changes do they make?)**

- How did you use your continuous glucose monitor and its data?
  What information on the app did you use most often?
- How, if at all, did your CGM data affect how you thought about food and the food choices you made?
  Did seeing your glucose information cause you to change the amount, type, timing, or something else about the foods you ate? What changes did you make? What did you eat more of or less of?
  Did you try any yes/less choices (Nutrition Guidance) to help reach your glucose targets? Why or why not?
  What made it hard to use your CGM numbers to make decisions about your food? What would have made it easier to use your CGM to guide your food choices?

**Cooldown**

- What else do you want to share about your experience learning how to use information from your CGM, or about how you now think about food choices with diabetes?

### Qualitative Data Analysis

Interviews were audio-recorded and transcribed using automated transcription software (Microsoft Teams). Interviewers took detailed field notes during the interview and memos [20] after the interview in a field note and memo guide in REDCap (Research Electronic Data Capture; Vanderbilt University) [21], which corresponded with the a priori coding tree. As interviews were completed, a lead qualitative analyst (MMJ) imported recordings, transcripts, field notes, and memos into qualitative analysis software (NVivo version 12; Lumivero). The lead analyst followed a sort-and-sift matrix analysis approach [22] to identify emergent themes within each research question and summarized key findings across interviews, identifying representative quotes. The analysis team (MMJ, HJW, MSGH, and LJZ) met for iterative reviews and to refine key findings. Although the concept of saturation does not directly translate to the rapid sort-and-sift matrix approach used in our study [23], analysts did consider the concept broadly and made note of when no new major themes emerged related to the a priori framework. This was done with issues of reflexivity in mind and to increase the correctness of findings [24]. Finally, a codebook and audit trail were maintained by the analysis team (MMJ, HW, MSGH, and LJZ) to ensure rigor and increase reproducibility.

Qualitative themes within each research question are presented along with representative quotes, which are embedded into the text to aid in the communication and richness of the findings described within each a priori construct in the coding tree [25]. Descriptive statistics, including means, SDs, frequencies, and percentages, are presented where appropriate. Participants did not review transcripts, codebooks, or other findings during or after analysis.

## Results

### Participant and Interview Characteristics

A total of 15 (88%) of the 17 eligible UNITE study participants agreed to participate in the qualitative interviews; 2 (12%) declined due to time constraints. Saturation was believed to be reached, as no new major themes emerged with iterative ongoing analysis. Most (13/15, 87%) interview participants identified as White and male (9/15, 60%). At the start of the UNITE study intervention, participants had a mean age of 64 (SD 7.4) years, had T2D for 7.5 (SD 3.8) years, had an HbA_1c_ of 7.5% (SD 0.4%), and had a TIR of 51% (SD 25%; Table 1). Interviews lasted an average of 31 (SD 5) minutes and were conducted between September 2023 and March 2024.

**Table 1:** Descriptive participant data (N=15).

Characteristics			Values
**Self-identified as male, n (%)**			9 (60)
**Age (y), mean (SD)**			64.2 (7.4)
**Racial or ethnic group, n (%)^a^**			
	African Native; American Indian or Alaskan Native; Asian (including Hmong, Chinese, Asian Indian, Vietnamese, etc); Black or African American; Hispanic or Latino, Latina, or Latinx; Middle Eastern or North African; or Native Hawaiian or Other Pacific Islander	1 (7)	
	White	13 (87)	
	Chose not to answer	1 (7)	
**Duration since T2D^b^ diagnosis (y), mean (SD)**			7.5 (3.8)
**Usual finger stick frequency at baseline, n (%)**			
	Never or less than once per month	3 (20)	
	1-3 times per month	3 (20)	
	1-6 times per week	4 (27)	
	Once per day	4 (27)	
	2-4 times per day	1 (7)	
**Food secure, n (%)^c^**			14 (93)
**Baseline HbA_1c_^d^ (%), mean (SD)**			7.5 (0.4)
**Baseline time in range (%; time with glucose 70-180 mg/dL), mean (SD)**			51 (25)

^a^Racial and ethnic groups were merged for data presentation to protect participant confidentiality.

^b^T2D: type 2 diabetes.

^c^Food security was confirmed if there was a positive answer to either of the following two questions: (1) “Within the past 12 months, I worried whether my food would run out before I had money to buy more.” (2) “Within the past 12 months, the food I bought just didn’t last, and I didn’t have money to get more.”

^d^HbA_1c_: hemoglobin A_1c_.

### Results of Research Question 1: How Do People Who Participate in an NFA During CGM Initiation Describe This Experience (ie, Intervention Receipt)?

During the first CGM initiation session, the CGM sensor and its data were explained to participants as tools to help guide their food choices. Participants were oriented to the CGM data displayed on the G7 smartphone app and encouraged to know (and remember) their glucose targets.

Approximately 2 months after the original CGM initiation session, the qualitative interviews were conducted, and it was clear that participants understood their CGM data. Participants were able to fluently and easily discuss real-time glucose values and metrics such as TIR and average glucose with their interviewers. While there were nuanced differences in the reported use of the data across participants (described in subsequent sections), these new CGM users seemed to have no difficulty understanding the CGM data, glucose targets, or how to use them.

Most of the participants remembered the nutrition-focused CGM initiation materials, and they generally liked the content and format. They could describe the core concepts presented in the materials (eg, the *1, 2, 3* approach and *yes/less* framework) in simple terms, even if not using the specific terminology. Some participants were able to discuss the materials unprompted, while others needed a brief review of the materials:

[After a brief review of the materials] That 1, 2, 3 approach—about checking my glucose before I eat, note what I ate, then note what happened after I ate—that became the real solid basis of my first two or three weeks with the monitor. It really helped me change my diet and I saw some pretty immediate benefits.

Participant #3

The nutrition information presented within the materials was recognized by participants as consistent with prior nutrition-related education. This repetition was not seen as negative, and some viewed it as a strength. Several participants commented that the plated food images and the message of “half the plate as vegetables” along with the CGM data were helpful for guiding adjustments to their food intake:

In fact, I was even thinking a little bit about it this morning, the pictures of the plate, the plate method did stick with me. That helped...The actual pictures of plates and having non-starchy vegetables as half and then a quarter protein, that was useful.

Participant #1

Many participants specifically noted liking the simplicity of the messages around using CGM data to understand the impact of food choices on their glucose numbers and the utility of having flexible glucose targets (eg, glucose 70-180 mg/dL and >70% TIR). No substantial suggestions to improve the content or format of the nutrition intervention materials were provided. One participant described the materials as “highly polished,” and many described the pictures and images as supporting their understanding of what to do with CGM data and food choices:

I’ve made a few PowerPoint presentations in my time, and I’d say these are very good, very, very good...the most educational part of the slide set was about the quantity and choices for what foods to eat; the fact that they talked about it at all, because I don’t pay attention to that. I have the foods that I like, and I think I know enough about them to know whether I’m having a good food or not...So, I would say being more aware of high-sugar foods and trying to minimize them [was a helpful message in the slides].

Participant #6

Participants described the 2 sessions with their diabetes care provider (the in-person initiation and remote check-in) as positive and useful and described the care providers as pleasant, kind, respectful, clear, knowledgeable, and thorough. One participant described the time with the care provider as feeling “more like a conversation about my health” than being “talked at,” and another participant described their care provider as especially helpful in dealing with feelings of guilt and blame related to food and diabetes.

One participant described the content of the discussions with the care provider as consistent with prior experiences but the tone as being distinctly more respectful, positive, and motivating. Another felt part of their success in using the CGM device to guide food choices was due to the consistent messaging from the diabetes care provider throughout the intervention period. However, others suggested that additional planned follow-up sessions with the diabetes care providers would have added benefits (ie, more appointments for CGM data review and discussion):

Checking in and reinforcing or affirming more [would have been useful during the program]. Maybe nudging and encouraging more health choices, because there’s a lot of emotional and cultural baggage that people have with foods you know, and it’s not an easy thing to change.

Participant #5

Overall, participants agreed that focusing on nutrition and food choices was a good way to help someone with T2D initiate CGM use and that this approach was beneficial for their diabetes care. Several participants specifically shared their appreciation for both the nutrition-focused intervention materials and the time with the diabetes care provider:

[In the past] I saw a nutritionist and it didn’t help me—and, I was given a glucose meter and it didn’t help me...But, the combination of that real-time glucose and then getting the tips [from the care provider] on what to try...it’s like, yes, what they’re telling me, now I can see it works!

Participant #14

Many described starting to use a CGM and considering their food and nutrition choices as essential. For participants who felt they were knowledgeable about nutrition before the intervention, they presumed they would have naturally thought about food choices when initiating CGM; however, this is challenging to ascertain, especially as it relates to consideration for food choices that align with evidence-based guidelines:

Well, nutrition, exercise, and medication is what I would consider to be the triangle. You have to [have these] to be successful...[Use of the CGM without the nutrition guidance] would not have been as good, not as effective...the effectiveness of the control of the blood sugar would have been less.

Participant #6

### Results of Research Question 2: How Do People Who Participate in an NFA During CGM Initiation Use CGM Data to Make Food-Related Decisions, and What Food-Related Changes Do They Make (ie, Intervention Enactment)?

All participants described regularly (eg, multiple times per day) using the G7 app to follow their glucose after the initial CGM initiation session. Difficulty using or interpreting CGM data was rarely described. Participants explained using CGM data both retrospectively (ie, the 3 or 14-day TIR) and in real time (ie, the glucose bubble, arrow, and 3-, 6-, 12-, or 24-hour glucose trend lines). Several participants expressed specific appreciation for the new diabetes management concepts, such as CGM-derived average glucose and TIR, and they described using these as guides for their diabetes care:

I thought that it was interesting where the time in range was. It helped me understand what you’re specifically looking at...I paid attention to it all the time.”

Participant #4

Many talked about following glucose levels before and after meals and activity, as recommended by the intervention’s *1, 2, 3* approach. However, some described “checking it all the time” or looking at the app “obsessively.” Participants described using the CGM data to make decisions in real time, and some described relying on the trend arrows as a way to make decisions about what to eat in the moment:

If I’m about to have dinner and [my numbers were near the top of the range] I would make different decisions about either what to eat or how much to eat...I might have a little less, or something that was lower carb, or definitely start with vegetables first. –

Participant #1

Many participants described using the recommended techniques (eg, the *1, 2, 3* approach) to learn how various foods and meals affected their glucose. Participants described experimenting to see the impact of *yes* foods (eg, nonstarchy vegetables), and 1 participant described trying various food substitutions to come up with a meal plan that worked well for their glucose management:

While it’s very helpful to see the numbers on your CGM, knowing more about how food impacts those numbers is so helpful...[I can see] if I fill up on vegetables my numbers will stay more consistent and/or lower...and, I swear that I enjoy my salads a lot more now...I found more satisfaction with my vegetables.

Participant #9

Many also described experimenting with *less* foods (eg, starchy snacks or sweetened foods) to learn how those foods affected their glucose levels. Some described the results of this type of experimentation as “surprising,” specifically noting they learned how long their glucose stayed elevated after eating foods they considered small “cheats” or “slipups.” Others also described using experimentation with *less* foods as an “excuse” to eat these foods “guilt free:”

I just ate things like a peanut butter and jelly sandwich or chocolate milk and, wow, for me drinking milk really makes the blood sugar go up. That was a sad thing to learn because I love drinking milk.

Participant #13

Others described experimenting with the timing and portion sizes of meals, including smaller meals throughout the day, delaying or skipping meals, trying to eat more protein before bed, or adding in physical activity throughout the day, especially right after meals. For most, experimentation with foods led to new perspectives and knowledge about the impact of foods and activity on glucose.

One participant described learning from her CGM data that allowing some feelings of hunger was “safe” for her diabetes management; in other words, she learned that hunger did not mean she needed to eat to prevent low glucose. For some, the increased knowledge and immediate feedback from experimentation led to changes in their perceptions of food, with a few describing a better appreciation for the value of foods. One participant described “losing the craving” for *less* foods because they were not “worth it”; for this participant, they described attaching more value to *yes* foods because they saw the beneficial impact on glucose:

[The CGM] helped me appreciate the value of foods. I love carbohydrates and could eat bread and pastry all day long and it will have a bad impact on my blood sugar—an enormously bad impact. And I like sweets. If I indulge in a sweet, it was a real reminder that I may be loving this sensation in my mouth and whatever is going on in my brain chemistry, but I’m not doing my overall health any good...Then, similarly for vegetables, I’m not a real fan of vegetables. But, watching a really high fiber, high vegetable meal have a low impact on my blood sugar, I had a very tangible reminder that these things are actually good for me.

Participant #3

Most participants described making at least some dietary changes to positively impact their glucose, and they actively extended experimentation into efforts to maintain improved glycemia or TIR. There were some clear, broad-level changes to food choices or behaviors that emerged as common among participants (eg, eating more nonstarchy vegetables, reducing overall carbohydrates or sugars, and choosing smaller portions); however, these interviews also highlighted that changes to specific foods and other behaviors were nuanced and unique to the individual. Table 2 provides an individual-level summary of some of the main food-related changes and behavior strategies the participants reported using to improve their glucose.

For example, individual participants reported details, such as switching from oatmeal and bananas for breakfast to cottage cheese and strawberries, choosing roasted peanuts in the shell for a snack to slow eating, or relying on cauliflower crust for pizzas. One participant reported making substantial changes to the amount of food consumed, stating that since seeing the CGM data, “I eat about half as much food now.” Another reported using their CGM to “guide every decision about food” when first starting with the device and then coming up with a meal plan and using the CGM data to determine when or if more changes were needed.

Not all participants made substantial changes to their food choices or behaviors. Some described a gap between increased knowledge and their perceived or realized ability to make changes. One participant specifically mentioned foods related to holidays, traditions, and culture as being hard to change even when seeing the CGM data. This seemed to pair with a few participants self-describing themselves as “poor eaters” or having negative opinions about their own eating patterns. While infrequent, it is also important to note that some people described CGM as amplifying feelings of needing to “try harder” and noted that CGM added stress because it was hard to avoid seeing the impacts of certain foods when the device “was always measuring me.” One person reported not liking the amount of mental energy they spent thinking about glucose and food; therefore, they ended up returning to old food habits:

It was always in my head that my blood sugar was always high even when it was at its lowest; it was still too high. So when I ate it would just be way too high...it kinda made me afraid to eat.

Participant #2

**Table 2:** Examples of the individual-level food and behavior changes participants described implementing after seeing their continuous glucose monitoring (CGM) data.

ID	Food changes	Behavior changes
1	More: nonstarchy vegetables, other vegetables, and melon Less: rice	Chose overall lower carbohydrates, ate vegetables before eating other foods, chose smaller portions, chose smaller meals spaced more evenly throughout the day, stopped eating before feeling full, skipped meals, and walked frequently (sometimes as much as every hour)
2	More: roasted peanuts in a shell Less: rice (smaller portions), mini-candy bars, and candy	Chose smaller portions and added activity after meals
3	More: cottage cheese and strawberries, large salads, leafy greens, fish, nuts, vegetables, and protein foods Less: oatmeal and grapes	Chose smaller portions, delayed evening meals, ate very low carbohydrate dinners, and walked in the afternoon
4	More: vegetables and homemade nonprocessed foods Less: fast food; sweets; and chocolate kisses	None noted
5	More: salads, peanut butter, sweet potato, and cauliflower crust for pizza Less: rice, crackers, chips, bread, Italian pasta, and alcohol	Measured portions, chose smaller portions overall (eg, half as much food), chose smaller portions of carbohydrates (eg, 1 piece of bread instead of 2), skipped meals, and walked more (even if only 10 min)
6	More: whole-wheat bread, whole-wheat pasta, and white meat Less: Soda, fruit juices, candy, and chocolate bars	None noted
7	More: none noted Less: cereal and bread	Chose smaller portions and walked more
8	More: Green leafy vegetables, other vegetables, fresh fruit, fresh whole foods, and low-sugar yogurt Less: candy, pure sugar foods, and chips	Chose smaller portions and chose lower carbohydrate options
9	More: water, black coffee, vegetables, salads, cucumbers, celery, eggs, popcorn, and protein foods Less: cereals	Chose smaller portions, reduced carbohydrate-heavy meals, delayed mealtimes, and walked after meals when glucose was high
10	More: vegetables and fruit Less: certain carbohydrates and certain types and amounts of cereals	Measured out servings, chose smaller portions (eg, half bagel instead of whole), and chose overall lower carbohydrate
11	More: no specific changes were noted; however, the participant reported confidence in using the CGM data and described examples of food experimentation Less: nothing noted	None noted
12	More: water Less: sweets	Chose smaller portions
13	More: several vegetables Less: milk	Chose smaller portions, chose overall lower carbohydrate (eg, dropped the bun), and ate a small amount of protein before bed
14	More: nonstarchy vegetables (steamer bags), cottage cheese, and protein foods Less: soda	Chose smaller portions, added more protein to meals, read food labels, and limited sweets and sugars
15	More: no changes were noted; however, the participant reported confidence in using the CGM data and reported several examples of current food choices that were reinforced because of seeing CGM data Less: cereals, pancakes, and baking with regular flour	None noted

In contrast, many participants described the CGM as finally providing them with a clear understanding of how their food choices influenced their glucose levels and diabetes, which in turn led to potentially more sustainable behavior changes. One participant described the impact of their participation in this NFA as something that helped them make changes in their diabetes management that they had not been able to do for years and another expressed excitement in seeing progress:

It helped me set a different pattern on when I ate, how much I ate, what I ate—those are changes I was unwilling to make until I saw the data.

Participant #4

This is the first time in 10 years that I’ve made progress!

Participant #14

Similarly, others described the CGM data as “encouraging to see how much control I had” and a way to see the impact of foods with new clarity:

I think focusing on nutrition is helpful for someone to get started using a CGM. It hit home that the choices I was making, like in crystal clear clarity, if I eat this, this happens, that happens. With the monitor, it showed it goes up this much.

Participant #2

Other participants shared special appreciation for the biofeedback following food choices, with one person describing the feedback loop as a “gamechanger” and another especially liking the immediacy of the data:

...many people intellectually understand nutrition, but don’t comply—the sensor is an immediate and absolute reminder of the changes and differences that [foods] make.

Participant #12

When participants were prompted for suggestions to improve the overall NFA, 1 (7%) of the 15 participants suggested pairing the CGM with structured meal plans, such as instructions for what to try eating for a week for improved glucose. Other suggestions focused more on ideas to improve the CGM app, such as a quick and easy way to record a meal in the app or to overlay their food notes with their glucose values. A participant suggested they would have liked it if the NFA intervention materials “were built into the app” for easier reference:

If there was a really convenient way to record what I was eating and have that tied very directly and very visibly to what the CGM app was showing me, that would have been hugely impactful.

Participant #3

## Discussion

### Principal Findings

Through these qualitative interviews, we heard that using an NFA during CGM initiation was generally well received and perceived as helpful for people with T2D who do not use insulin. We also found that in this population of people who do not use insulin and who infrequently monitored glucose (with finger sticks), the CGM data were easily understood, regularly viewed, and often used to promote changes in food choices and behaviors during the 2-month study. The nutrition-focused intervention materials and messages were mostly described as supportive and useful for helping participants understand how to use their CGM data to guide food choices.

### Relationship to Prior Work

The results of this research add to existing literature in several ways, including highlighting how the CGM device could potentially be used to specifically encourage evidence-based nutrition recommendations. Research demonstrates that choosing high-quality eating patterns (ie, adhering to evidence-based nutrition recommendations) is linked to better glycemia [26,27] and inversely associated with risk of all-cause mortality, cardiovascular disease, cancer, and neurodegenerative diseases [28]. Therefore, any diabetes technology or care approach that can integrate messages about the importance of diet quality could be of significant benefit. These interviews not only reinforced the notion that there is no one-size-fits-all diet or lifestyle plan that works for everyone with diabetes [2] but also that CGM can be used to help individuals identify which specific foods and behavior strategies work best for them. Findings from this research may also help support the conclusions of previous research, which have suggested that CGM can lead to lifestyle and behavior changes [29-31] but where objective behavior outcomes were not measured or qualitatively assessed.

In addition, this research provides context regarding opportunities for training new CGM users on optimal use of the device. The American Diabetes Association’s Standards of Care recommend that education, training, and ongoing support are needed for all diabetes devices, including continuous glucose monitors [5]. Furthermore, Heinemann and Klonoff [32] expanded upon how CGM use in and of itself does not necessarily lead to better outcomes (ie, improved glucose), which may be particularly true for people with T2D who do not take insulin and are less reliant on (or familiar with) glucose testing. At the same time, lack of nutrition guidance and support has also been identified as a prominent barrier to behavior change for people with T2D [33]. Thus, education about how to optimally interpret and use CGM data, specifically to guide food choices that align with evidence-based guidance, seems of benefit. Our interviews suggest that using an NFA during CGM initiation could be a helpful way to both educate on the device and its data and empower new users to use the data to make healthful adjustments to their food choices and behaviors. With this NFA, participants seemed to have little to no difficulty interpreting CGM metrics and using them to guide food choices, which suggests that providing education on both glycemic targets and evidence-based eating principles (at the same time) during CGM initiation is reasonable.

Related to CGM support, it is important to note that this intervention provided to these new CGM users was very brief—just 1 in-person session and 1 remote follow-up session approximately 14 days later. Some participants suggested that more follow-up sessions would have been beneficial. The need for additional follow-up sessions aligns with recommendations for adequate diabetes self-management education [2] and with recent research suggesting that—based on individual circumstances and goals—evolving support is needed to sustain effective CGM use [34]. At this time, it is unclear how often CGM data reviews are needed to support glycemic goal attainment or maintenance, and therefore, further research is needed. Future research should aim to help define best practices for the ideal frequency of health care provider–led CGM data review, for the most efficient ways to systematically and effectively assess and discuss CGM data with users, and for using CGM data specifically as a tool to help sustain long-term lifestyle and behavior change.

The results of this research provide the diabetes care community with considerations for how to present or position nutrition messages when initiating CGM in people with T2D.

These interviews suggest that using a positive, respectful tone to discuss evidence-based nutrition guidance during CGM initiation was beneficial; however, we also encountered the potential for CGM data to exacerbate negative feelings about oneself or one’s relationship with food. Some people described skipping or delaying meals as a means to try and stay in range, which could be acceptable or could be concerning, for example in people with a history of or potential for disordered eating [35]. Others expressed fears and frustration over thinking too much about their CGM data as it related to foods. Taken together, this underscores the importance of ensuring that CGM education includes messaging about how foods and behaviors are only part of what drives glycemia, especially for people who are not using CGM primarily to determine medication doses or adjustments. On the basis of this research, it seems important for diabetes care providers to regularly remind CGM users that sometimes even with the best adherence to nutrition or lifestyle plans, additional medication support may be needed. In other words, diabetes care providers should make it clear that the CGM device is meant to be a support (eg, for positive nutrition and lifestyle changes and medication management), and it should not contribute to negative feelings, stress, or disordered eating. These concepts can be considered further by exploring previous qualitative research describing the psychosocial outcomes [36], quality of life [37], and other attitudes and behaviors [38] of people with T2D using CGM.

### Strengths and Limitations

This research has several strengths and limitations. The first strength is the qualitative assessment of people who underwent a well-defined intervention that was designed specifically for the purposes of using CGM to guide evidence-based nutrition and lifestyle choices. The second strength is the methodology used to design, conduct, and analyze these interviews. Furthermore, the third strength is that this work focused exclusively on CGM initiation in people with T2D who do not use insulin, as people with T2D who do not use insulin and who use CGM is a segment of the diabetes population that has been evaluated less frequently than others.

Regarding limitations, the first limitation is that this research did not assess the perspectives and behaviors of people with T2D who initiated CGM without an NFA (eg, with an SDA). Thus, it is unclear whether people without an NFA during CGM initiation would have similar experiences and report similar changes or whether they would consider the importance of nutrition choices for other aspects of health; future research should consider this. Second, the participants interviewed were predominantly White (13/15, 87%), food secure (14/15, 93%), and identified as males (9/15, 60%) with a lower HbA_1c_ at baseline, which may limit the generalizability of the findings because we cannot account for how the nutrition-focused intervention materials would be received by a more diverse audience (eg, food images and core messaging). It is possible the materials would be more or less applicable based on recipient characteristics, and further research in a more diverse population is needed. Third, while this research describes the participants’ reports of their CGM use and their nutrition and lifestyle behaviors over a 2-month period, these behaviors were not objectively measured or connected to the participants’ actual glycemic outcomes. However, these objective data will be available with the results of the larger UNITE study.

### Future Research

Future research should assess the experiences and behaviors of people with diabetes who participate in an NFA intervention over a longer period and with more health care provider–led CGM data reviews or could explore factors that may contribute to negative experiences or stress around using CGM data to guide food and lifestyle changes.

### Conclusions

First-line therapy for T2D management is lifestyle modification, which includes following evidence-based nutrition guidelines and increasing physical activity. CGM data can be used to promote or encourage these lifestyle changes. This qualitative study described the experiences and reported behavioral effects of using an NFA during CGM initiation in people with T2D who were not using insulin.

Approximately 2 months after initiating CGM using an NFA (which included 1 in-person and 1 remote follow-up session), participants seemed to clearly understand the meaning and application of CGM data for behavior change. They reported using their real-time and retrospective CGM data regularly, and they agreed that pairing evidence-based nutrition information with CGM initiation instructions was helpful for their diabetes care. Most participants reported making some food and behavior changes that aligned with evidence-based guidance for a healthy lifestyle, such as increasing nonstarchy vegetable intake or decreasing overall sugar intake. At the individual level, participants also noted several unique food or behavior changes, which highlights that no single eating plan works for all people with diabetes but that CGM can likely show which eating plan may work best for an individual. Opportunities exist to further explore best practices for CGM-guided nutrition interventions in people with diabetes.

## Appendix

Multimedia Appendix 1 UNITE (Using Nutrition to Improve Time in Range) study qualitative interview guide.

## Abbreviations

CGM: continuous glucose monitoring
COREQ: Consolidated Criteria for Reporting Qualitative Research
HbA_1c_: hemoglobin A_1c_
NFA: nutrition-focused approach
REDCap: Research Electronic Data Capture
SDA: self-directed approach
T2D: type 2 diabetes
TIR: time in range
UNITE: Using Nutrition to Improve Time in Range

## Data Availability

The datasets generated during and analyzed during this study are available from the corresponding author on reasonable request.

## References

[1] American Diabetes Association Professional Practice Committee (2024). 9. pharmacologic approaches to glycemic treatment: standards of care in diabetes-2024. Diabetes Care.

[2] American Diabetes Association Professional Practice Committee (2024). 5. facilitating positive health behaviors and well-being to improve health outcomes: standards of care in diabetes-2024. Diabetes Care.

[3] Ajjan RA, Battelino T, Cos X, Del Prato S, Philips JC, Meyer L, Seufert J, Seidu S (2024). Continuous glucose monitoring for the routine care of type 2 diabetes mellitus. Nat Rev Endocrinol.

[4] Evert AB, Dennison M, Gardner CD, Garvey WT, Lau KH, MacLeod J, Mitri J, Pereira RF, Rawlings K, Robinson S, Saslow L, Uelmen S, Urbanski PB, Yancy WS (2019). Nutrition therapy for adults with diabetes or prediabetes: a consensus report. Diabetes Care.

[5] American Diabetes Association Professional Practice Committee (2024). 7. diabetes technology: standards of care in diabetes-2024. Diabetes Care.

[6] Barnard-Kelly KD, Polonsky WH (2020). Development of a novel tool to support engagement with continuous glucose monitoring systems and optimize outcomes. J Diabetes Sci Technol.

[7] Isaacs D, Cox C, Schwab K, Oser TK, Rinker J, Mason MJ, Greenwood DA, Albanese-O'Neill A (2020). Technology integration: the role of the diabetes care and education specialist in practice. Diabetes Educ.

[8] Lind N, Christensen MB, Nørgaard K (2024). A combined diabetes and continuous glucose monitoring education program for adults with type 2 diabetes. PEC Innov.

[9] Powers MA, Bardsley JK, Cypress M, Funnell MM, Harms D, Hess-Fischl A, Hooks B, Isaacs D, Mandel ED, Maryniuk MD, Norton A, Rinker J, Siminerio LM, Uelmen S (2020). Diabetes self-management education and support in adults with type 2 diabetes: a consensus report of the American Diabetes Association, the Association of Diabetes Care and Education Specialists, the Academy of Nutrition and Dietetics, the American Academy of Family Physicians, the American Academy of PAs, the American Association of Nurse Practitioners, and the American Pharmacists Association. Diabetes Care.

[10] Chatterjee S, Davies MJ, Heller S, Speight J, Snoek FJ, Khunti K (2018). Diabetes structured self-management education programmes: a narrative review and current innovations. Lancet Diabetes Endocrinol.

[11] Polonsky WH, Fortmann AL, Soriano EC, Guzman SJ, Funnell MM (2023). The AH-HA! project: transforming group diabetes self-management education through the addition of flash glucose monitoring. Diabetes Technol Ther.

[12] Willis HJ, Johnson E, JaKa M (2024). A nutrition-focused approach during continuous glucose monitoring initiation in people with type 2 diabetes: using a theoretical framework to unite continuous glucose monitoring and food choices. J Diabetes Sci Technol.

[13] Vindrola-Padros C, Johnson GA (2020). Rapid techniques in qualitative research: a critical review of the literature. Qual Health Res.

[14] Watkins DC (2017). Rapid and rigorous qualitative data analysis: the “RADaR” technique for applied research. Int J Qual Methods.

[15] Azungah T (2018). Qualitative research: deductive and inductive approaches to data analysis. Qual Res Psychol.

[16] Bellg AJ, Borrelli B, Resnick B, Hecht J, Minicucci DS, Ory M, Ogedegbe G, Orwig D, Ernst D, Czajkowski S (2004). Enhancing treatment fidelity in health behavior change studies: best practices and recommendations from the NIH Behavior Change Consortium. Health Psychol.

[17] Tong A, Sainsbury P, Craig J (2007). Consolidated criteria for reporting qualitative research (COREQ): a 32-item checklist for interviews and focus groups. Int J Qual Health Care.

[18] Baker SE How many qualitative interviews is enough? Expert voices and early career reflections on sampling and cases in qualitative research. National Centre for Research Methods.

[19] Adams WC, Newcomer KE, Hatry HP, Wholey JS (1995). Conducting semi-structured interviews. Handbook of Practical Program Evaluation.

[20] Razaghi N, Abdolrahimi M, Salsali M (2008). Memo and memoing in qualitative research: a narrative review. J Qual Res Health Sci.

[21] Harris PA, Taylor R, Minor BL, Elliott V, Fernandez M, O'Neal L, McLeod L, Delacqua G, Delacqua F, Kirby J, Duda SN (2019). The REDCap consortium: building an international community of software platform partners. J Biomed Inform.

[22] Maietta R, Mihas P, Swartout K, Petruzzelli J, Hamilton A (2021). Sort and sift, think and shift: let the data be your guide an applied approach to working with, learning from, and privileging qualitative data. Qual Res.

[23] Higham R, Pini S, Quyn A, Kowal M, Helliwell J, Saman R, Lewthwaite P, Young N, Rousseau N (2022). Rapid qualitative analysis in a mixed-methods evaluation of an infection prevention intervention in a UK hospital setting during the COVID-19 pandemic: a discussion of the CLEAN study methodology. Front Sociol.

[24] May T, Perry B (2024). The SAGE Handbook of Qualitative Data Analysis.

[25] Eldh AC, Årestedt L, Berterö C (2020). Quotations in qualitative studies: reflections on constituents, custom, and purpose. Int J Qual Methods.

[26] Gradinariu V, Ard J, van Dam RM (2023). Effects of dietary quality, physical activity and weight loss on glucose homeostasis in persons with and without prediabetes in the PREMIER trial. Diabetes Obes Metab.

[27] Antonio JP, Sarmento RA, de Almeida JC (2019). Diet quality and glycemic control in patients with type 2 diabetes. J Acad Nutr Diet.

[28] Morze J, Danielewicz A, Hoffmann G, Schwingshackl L (2020). Diet quality as assessed by the healthy eating index, alternate healthy eating index, dietary approaches to stop hypertension score, and health outcomes: a second update of a systematic review and meta-analysis of cohort studies. J Acad Nutr Diet.

[29] Aleppo G, Beck RW, Bailey R, Ruedy KJ, Calhoun P, Peters AL, Pop-Busui R, Philis-Tsimikas A, Bao S, Umpierrez G, Davis G, Kruger D, Bhargava A, Young L, Buse JB, McGill JB, Martens T, Nguyen QT, Orozco I, Biggs W, Lucas KJ, Polonsky WH, Price D, Bergenstal RM (2021). The effect of discontinuing continuous glucose monitoring in adults with type 2 diabetes treated with basal insulin. Diabetes Care.

[30] Aronson R, Brown RE, Chu L, Bajaj HS, Khandwala H, Abitbol A, Malakieh N, Goldenberg R (2023). IMpact of flash glucose monitoring in pEople with type 2 diabetes inadequately controlled with non-insulin Antihyperglycaemic ThErapy (IMMEDIATE): a randomized controlled trial. Diabetes Obes Metab.

[31] Layne JE, Jepson LH, Carite AM, Parkin CG, Bergenstal RM (2024). Long-term improvements in glycemic control with Dexcom CGM use in adults with noninsulin-treated type 2 diabetes. Diabetes Technol Ther.

[32] Heinemann L, Klonoff DC (2020). An opportunity to increase the benefit of CGM usage: the need to train the patients adequately. J Diabetes Sci Technol.

[33] Montilva-Monsalve J, Dimantas B, Perski O, Gutman LM (2023). Barriers and enablers to the adoption of a healthier diet using an app: qualitative interview study with patients with type 2 diabetes mellitus. JMIR Diabetes.

[34] Mayberry LS, Nelson LA, Bergner EM, Raymond JK, Tanenbaum ML, Jaser SS, Wiebe DJ, Allen N, Berg CA, Naranjo D, Litchman M, Ollinger L, Hood K (2024). Time for a reframe: shifting focus from continuous glucose monitor uptake to sustainable use to optimize outcomes. J Diabetes Sci Technol.

[35] Wallace T, Heath J, Koebbel C (2023). The impact of flash glucose monitoring on adults with type 1 diabetes' eating habits and relationship with food. Diabetes Res Clin Pract.

[36] Soriano EC, Polonsky WH (2023). The influence of real-time continuous glucose monitoring on psychosocial outcomes in insulin-using type 2 diabetes. J Diabetes Sci Technol.

[37] Polonsky WH, Soriano EC, Fortmann AL (2022). The role of retrospective data review in the personal use of real-time continuous glucose monitoring: perceived impact on quality of life and health outcomes. Diabetes Technol Ther.

[38] Clark TL, Polonsky WH, Soriano EC (2024). The potential impact of continuous glucose monitoring use on diabetes-related attitudes and behaviors in adults with type 2 diabetes: a qualitative investigation of the patient experience. Diabetes Technol Ther.

