# Supplementary material for: “Now I can see it works!” Perspectives on Using a Nutrition-Focused Approach When Initiating Continuous Glucose Monitoring in People with Type 2 Diabetes: Qualitative Interview Study
Source: JMIR Diabetes. 2025 Jan 10;10:e67636. doi: 10.2196/67636 (PMC11759913; doi:10.2196/67636)
Supplement: Multimedia Appendix 1 [file diabetes_v10i1e67636_app1.docx]

**Supplemental Material**

UNITE study

Qualitative Interview Guide

**Interview Questions**

Root 1: First, can you tell me what you remember about when you were first diagnosed with diabetes?

- How did you take care of your diabetes at that time?
- Did you use a fingerstick glucose meter to check your glucose numbers (blood sugar)?
- Did you think about nutrition or food choices at that time?
- Did you ever talk with a diabetes educator or dietitian? Tell me about that experience.

Root 2: What do you remember talking about with your care provider during the appointment the day you first started using your CGM?

- What did you think about the information they gave you and how it was presented?
- When you first started using your CGM, your diabetes educator might have used some interactive slides on a computer screen to show you how to use your CGM. There is a copy of these slides to help you remember. Take a look at the packet with the letter A on the cover.
  - What did you think of this? What did you like? What didn’t you like?
  - Did the images in the slides help you know how to use your new CGM to make food choices? Tell me more. Can you give me a specific example of something you liked or that was helpful?
  - What ideas do you have for improving the interactive slides and/or the visuals?
- During the visits with your educator, you might have used a CGM Nutrition Guide. There is also a copy of this guide in the room to help you remember. Take a look at the packet with the letter B on the cover.
  - What did you think of this guide? What did you like? What didn’t you like?
  - Did the guide help you to know how to use your new CGM to make food choices? How might your experience have been different if you didn’t have this guide? Tell me more. Can you give me a specific example of something that was helpful or not?
  - Did the guide help you think about how you could use your food choices to improve your glucose and take care of your diabetes? Tell me more. Can you give me a specific example of something you liked or that was helpful?
  - What ideas do you have for improving this guide?
- Do you think focusing on nutrition is a good way to help someone get started using their CGM? Why or why not?

Root 3: Can you tell me about how you used your continuous glucose monitor during the UNITE study?

- What information from your G7 or Clarity app did you use most often?
  - For example, did you use the glucose bubble with the trend arrow, the hourly glucose graph, or the Time in Range bar? What about the notes section or the alerts?
- How did you use the information from the app? What was your thought process once you had this information?
- Can you give me a specific example of seeing your glucose information and what you thought about when you saw it? Tell me more.

Root 4: How, if at all, did your CGM data affect how you thought about food?

- Can you give me an example?
- Did seeing your glucose information cause you to change the amount, type, timing, or something else about the foods you ate? What foods and in which direction? Tell me more. Anything else you can share?
  - What did you find yourself eating more of?
  - What did you find yourself eating less of?
- Did you try eating different foods while wearing the CGM to see how they impacted your glucose or your Time in Range? Why or why not?
  - [If yes] What did you try? What surprised you?
  - Did you try using any of the YES/LESS suggestions to improve your glucose? Why or why not?
- What made it hard for you to use your CGM data/numbers to make decisions about your food?
- Can you think of anything that would make it easier for you to use your CGM data to make decisions about what to eat?

Root 5: What else do you want to share about your experience learning how to use information from your CGM or about how you now think about food choices with diabetes?
